# Modulation of Inflammatory Response in a Cirrhotic Rat Model with Induced Bacterial Peritonitis

**DOI:** 10.1371/journal.pone.0059692

**Published:** 2013-03-20

**Authors:** Elisabet Sánchez, Rubén Francés, Germán Soriano, Beatriz Mirelis, Francesc J. Sancho, José Manuel González-Navajas, Carlos Muñoz, Xiao-yu Song, Miguel Pérez-Mateo, José Such, Carlos Guarner

**Affiliations:** 1 Liver Section, Department of Gastroenterology, Hospital de la Santa Creu i Sant Pau, Barcelona, Spain; 2 Centro de Investigación Biomédica en Red en el Área temática de Enfermedades Hepáticas y Digestivas (CIBERehd), Instituto de Salud Carlos III, Madrid, Spain; 3 Institut dInvestigacions Biomédiques de Sant Pau (IIB), Barcelona, Spain; 4 Universitat Autònoma de Barcelona, Bellaterra (Cerdanyola del Vallès), Spain; 5 Department of Microbiology, Hospital de la Santa Creu i Sant Pau, Barcelona, Spain; 6 Department of Pathology, Hospital de la Santa Creu i Sant Pau, Barcelona, Spain; 7 Unidad Hepática, Hospital General Universitario, Universidad Miguel Hernández, Alicante, Spain; 8 Department of Microbiology, Hospital General Universitario, Universidad Miguel Hernández, Alicante, Spain; 9 Research and Development Centocor, Malvern, Pennsylvania, United States of America; Cordelier Research Center, INSERMU872-Team16, France

## Abstract

Bacterial peritonitis is a severe complication in patients with cirrhosis and ascites and despite antibiotic treatment, the inflammatory response to infection may induce renal dysfunction leading to death. This investigation evaluated the effect of TNF-α blockade on the inflammatory response and mortality in cirrhotic rats with induced bacterial peritonitis treated or not with antibiotics. Sprague-Dawley rats with carbon-tetrachloride-induced cirrhosis were treated with an intraperitoneal injection of 10^9^ CFU of *Escherichia coli* diluted in 20 mL of sterile water to induce bacterial peritonitis and randomized to receive subcutaneously-administered placebo, ceftriaxone, anti-TNF-α mAb and ceftriaxone, or anti-TNF-α mAb alone. No differences were observed between groups at baseline in respect to renal function, liver hepatic tests, serum levels of nitrite/nitrate and TNF-α. Treatment with ceftriaxone reduced mortality (73.3%) but differences did not reach statistical significance as compared to placebo. Mortality in rats treated with ceftriaxone and anti-TNF-α mAb was significantly lower than in animals receiving placebo (53% vs. 100%, p<0.01). Serum TNF-α decreased significantly in surviving rats treated with ceftriaxone plus anti-TNF-α mAb but not in treated with antibiotics alone. Additional studies including more animals are required to assess if the association of antibiotic therapy and TNF-α blockade might be a possible approach to reduce mortality in cirrhotic patients with bacterial peritonitis.

## Introduction

Spontaneous bacterial peritonitis (SBP) is a common and severe infection in patients with cirrhosis. Short-term prognosis has improved in recent decades due to prompt diagnosis during routine paracentesis [1], standardization of diagnostic criteria based on ascitic fluid analyses [2], [3], and use of non-nephrotoxic third generation cephalosporins [4]. However, a significant number of patients with SBP still develop complications such as infections, systemic hemodynamic dysfunction and progressive renal failure, that lead to death [1], [2]. Fifty percent of SBP patients who develop renal failure die during hospitalisation compared to only 6% of patients without this complication [5]. The administration of albumin to these patients has demonstrated a reduction in the incidence of renal dysfunction and improvement in short-term survival [5], [6].

Episodes of SBP are associated with a marked release of proinflammatory cytokines such as tumour necrosis factor alpha (TNF-α) and effector molecules like nitric oxide metabolites (NOx) that keep a close relationship with SBP-induced morbidity and mortality [7], [8]. Patients with SBP show a long-lasting marked increase in serum NOx that may contribute to maintaining splanchnic vasodilatation and thus worsen the hemodynamic hyperkinetic state [9], [10]. Besides, nitrite and nitrate levels in serum and ascitic fluid at diagnosis of infection are significantly higher in SBP patients who develop renal impairment as a consequence of the ascitic fluid infection than in patients who maintain a stable renal function [11].

Our group has recently reported that patients with SBP present recurrent episodes of bacterial translocation (BT) and maintain a marked inflammatory reaction [12] despite the administration of third generation cephalosporins. In rats, a new therapy with the blockade of TNF-α has two direct consequences: it blunts the development of the hyperdynamic circulation and reduces portal pressure in a model of portal hypertension [13], and reduces the frequency of BT episodes in model of cirrhosis [14]. Accordingly, the association of the usual third-generation cephalosporin with TNF-α blockade during a peritonitis episode may not only slow down the ongoing infection, but also improve survival. However, since TNF-α is part of the normal immune response, it is necessary to assess whether TNF-α blockade would increase the risk of developing superinfections.

We previously developed an experimental model of induced bacterial peritonitis in cirrhotic rats with or without ascites [15] that mimics SBP in patients, and considered it might be useful to evaluate the efficacy of new therapeutic interventions on short-term prognosis of patients with SBP. The present study aimed, therefore, to evaluate the effect of TNF-α blockade on the inflammatory response and mortality in cirrhotic rats with induced bacterial peritonitis treated or not with antibiotics.

## Materials and Methods

### Animals

Male Sprague-Dawley were purchase from Harlan Laboratories. Rats were individually caged at a constant room temperature of 21°C, exposed to a 12∶12 light/dark cycle and allowed free access to water and rat chow.

The study was approved by the Animal Research Committee at the Institut de Recerca of Hospital de la Santa Creu i Sant Pau (Barcelona) and by the Department of Agriculture, Livestock and Fisheries of the Generalitat de Catalunya (DARP). Animals received care according to the criteria outlined in the Guide for the Care and Use of Laboratory Animals.

### Induction of Cirrhosis

Cirrhosis was induced as previously described by Runyon et al [16]. Rats weighing 100-120 g were fed standard rodent chow (B/K) and were treated with 1.5 mM/L phenobarbital in tap water. When rats reached a weight of 200 g weekly doses of carbon-tetrachloride (CCl_4_) (J.T. Baker Inc., Phillipsburg, NJ) were given intragastrically using a sterile pyrogen-free syringe (Artsana p.p.a., Greenclate) with an attached stainless steel animal feeding tube (Popper and Sons, New Hyde Park, NY) without anaesthesia. The first dose of CCl_4_ was 20 µL and subsequent doses were adjusted based on changes in weight 48 hours after the last dose, as previously reported [17].

### Experimental Design

CCl_4_ was administered over 16 weeks, the required period for cirrhosis development [16]. To rule out the presence of ascites a paracentesis was performed under air anaesthesia with isofluorane (Forane, Abbott ind.). Sixty rats were then immediately injected intraperitoneally (i.p.) with a dose of 10^9^ colony-forming unit (CFU) of *Escherichia coli (E. coli)* diluted in 20 mL of sterile water to induce bacterial peritonitis as previously reported [15]. Four hours later, a blood sample was taken from the saphena vein, centrifuged and stored at −80°C for subsequent analysis.

Animals were then randomly allocated into four groups to receive: serum subcutaneously (s.c.) (Group I, n = 15), ceftriaxone 100 mg/kg s.c. daily for 7 days beginning 4 hours after *E. coli* injection (Group II, n = 15), anti-TNF-α mAb 15 mg/kg i.p. in a single dose (Centocor R&D, Inc, Malvern, PA, USA) and ceftriaxone 100 mg/kg s.c. daily for 7 days (Group III, n = 15) and monoclonal Antibody Anti-Tumour Necrosis Factor Alfa (anti-TNF-α mAb) 15 mg/kg i.p. in a single dose (Group IV, n = 15). Survival was monitored daily for 7 days.

### Laparotomy

Laparotomy was performed under anaesthesia with 10 mg/kg xylacine (Rompun, Bayer) and 50 mg/kg ketamine (ketolar, Parke-Dawis) in strictly sterile conditions in surviving rats 7 days after induction of peritonitis. In brief, abdominal fur was removed with a depilatory and the skin was sterilised with iodine. A short incision in the abdominal wall (3–4 cm.) was performed and a sample of intraperitoneal fluid was obtained for bacterial culture. The abdomen was then opened via a 3 cm median incision and the remaining fluid was evacuated. If no free fluid was present, sterile swabs were passed over the parietal peritoneal surface and then plated. Samples of pleural fluid were also collected for microbiological study. The mesenteric lymph nodes from the ileo-cecal area were aseptically dissected, removed, weighed, and liquefied in sterile saline for bacterial culture. Blood was collected from the cava vein in a non-additive sterile interior vacutainer (Becton Dickinson Vacutainer Systems Eur., Meylan Cedex, France), centrifuged and stored to determine liver and renal function parameters and TNF-α and NOx levels. A sample of the liver was also obtained for histological evaluation. All samples were stored at –80°C. Rats were then euthanized with intravenous sodium thiopentate (Penthotal, Abbott Laboratories).

### Biochemical Analysis

Immunoassays for quantitative measurement of rat TNF-α in blood samples were performed using TNF-α Quantikine rat Immunoassays (R&D Systems, Abingdon, UK) according to the manufacturer’s instructions. All samples were tested in duplicate and read at 450 nm and 490 nm in a ThermoMax microplate reader (Molecular Devices, Sunnyvale, California, USA).

The sum of the NOx: nitrite (NO_2_^−^) and nitrate (NO_3_^−^) is widely used as an index of NO generation and expressed as NOx levels [18]. NOx levels were calculated by measuring conversion of NO_3_^−^ to NO_2_^−^ by the enzyme nitrate reductase using an ELISA assay (R&D Systems, Minneapolis, MN) based on the Griess reaction that absorbs visible light at 540 nm, and expressed as µmol/L. All samples were tested in duplicate, and values were corrected by running samples with culture media to assess background NOx levels.

### Statistical Analysis of Experimental Data

Statistical analyses were performed with the SPSS statistical package (SPSS Inc. version 17.0, Chicago, Illinois, USA). All biochemical parameters are reported as mean ± SD. Differences between groups were analysed using the non-parametric Mann-Whitney U-test. Fisher’s exact test was used to compare mortality between groups. The Wilcoxon non-parametric test was used to compare variations of serum analytical parameters after inoculation with *E. coli*. A two-tailed p<0.05 was considered statistically significant.

## Results

**Figure 1:**
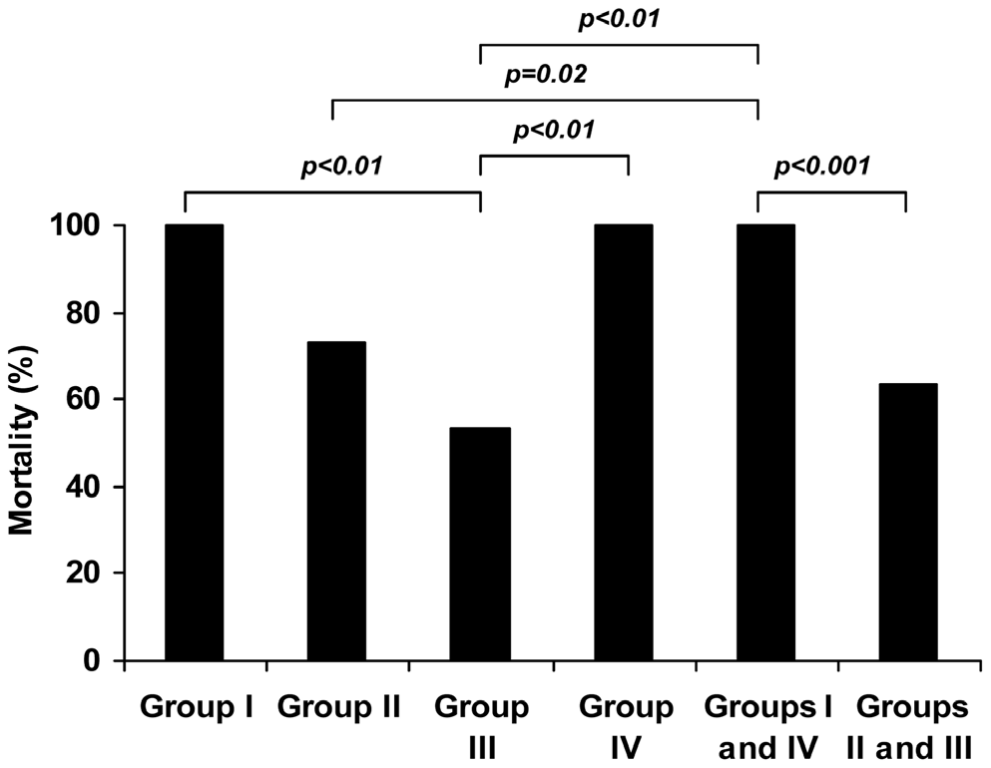
Mortality observed in rats with cirrhosis and induced bacterial peritonitis during one week after *E. coli* inoculation. Group I: placebo (serum s.c.); Group II: ceftriaxone s.c.; Group III: anti-TNF-α mAb i.p.+ceftriaxone s.c.; Group IV: anti-TNF-α mAb i.p.; Groups I and IV (non-antibiotic treated rats) and Groups II and III (antibiotic treated rats).

Sixty rats with cirrhosis and without ascites, as demonstrated by a negative diagnostic paracentesis, were included and randomised. Forty-nine rats (81**.**7%) died during the study, mainly during the first 24 hours after *E. coli* injection. No rats died in the first 4 hours after intraperitoneal *E. coli* inoculation. Figure 1 shows mortality during the week of the study. As detailed, all rats in groups I (placebo) and IV (treated with anti-TNF-α mAb alone) died during the study period. A significant reduction in mortality was observed in the overall group of cirrhotic rats treated with antibiotic with or without anti-TNF-α mAb (63.3%, groups II and III) as compared with animals not receiving antibiotics (100%, groups I and IV, p<0.001). Mortality in the group of animals treated with ceftriaxone alone (73.3%, group II) was lower than the corresponding value in groups I or IV, although differences did not reach statistical significance, probably due to the low number of animals tested. In contrast, infected cirrhotic rats treated with the combination of ceftriaxone plus anti-TNF-α mAb (group III) showed a significant reduction in mortality (53.3%) when compared to non-treated rats (group I, p<0.01) or to rats treated with anti-TNF-α mAb alone (group IV, p<0.01).

Table 1 shows basal analytical parameters, including renal and liver function tests and serum TNF-α and NOx levels, obtained four hours after *E. coli* injection. All parameters were similar in all four groups of cirrhotic rats included in the study. When comparisons were established between non-surviving *versus* surviving animals, basal serum NOx was the only analytical parameter showing a significant increase in non-surviving rats (p<0.05).

**Table 1:** Basal analytical parameters of the groups of cirrhotic rats with induced bacterial peritonitis.

GROUPS	I (n = 15)	II (n = 15)	III (n = 15)	IV (n = 15)	Non-surviving rats (n = 49)	Surviving rats (n = 11)
Urea (mM)	6,6±1,2	7,0±1,8	6,1±0,9	6,6±1,9	6,6±1,7	6,4±1,0
Creatinine (µM)	57,8±6,6	56,3±9,5	52,5±9,2	57,6±8,9	55,6±8,9	54,3±7,5
Bilirubine (µM)	11,9±9,5	12,5±7,2	17,1±10,1	13,6±10,2	15,1±9,6	12,9±7,7
AST (u/L)	1140±363	1020±585	1091±387	1014±616	1089±547	929±420
ALT (u/L)	622±316	693±482	608±263	640±509	650±438	640±349
GGT (u/L)	10,9±6,2	10,8±5,2	12,7±7,4	13,7±6,2	12,6±5,9	11,1±7,2
TNF-α (pg/mL)	176±100	163±96	204±118	207±137	198,6±117,7	167,6±101,4
NOx (nmol/mL)	107±30	98±39	121±46	129±89	125,4±62,0*	84,9±26,9

Samples of blood were obtained 4 hours after intraperitoneally administration of *E. coli.*

* p<0.05 respect to surviving rats.

Variations in renal and liver function tests and serum TNF-α and NOx levels were studied in surviving rats in samples obtained 4 hours after bacterial inoculation and at laparotomy. As detailed in Table 2, a significant reduction in TNF-α serum levels was observed in these rats (groups II+III, p<0.05). This variation was even more remarkable in rats treated with ceftriaxone plus anti-TNF-α mAb (p<0.05). The decrease of serum TNF-α levels in rats treated with antibiotic alone, however, did not reach statistical significance. NOx levels showed a tendency to increase at laparotomy in comparison with baseline values in all surviving animals but this was only statistically significant in Groups II+III (p<0.05). Liver parameters also decreased significantly in these rats (p<0.01) and no variations in renal function were observed.

**Table 2:** Variations in renal and liver function tests and serum TNF-α and NOx levels in surviving cirrhotic rats between the infection of *E.coli* and the laparotomy.

GROUPS	II (n = 4 )	III (n = 7 )	II+III (n = 11 )
Urea (mM)	6.7±1.0 → 6.3±0.9	6.2±1.0 → 6.1±0.4	6.4±1.0 → 6.1±0.6
Creatinine (µM)	51.5±4.1 → 51.3±3.9	56.0±8.8 → 56.3±7.6	54.3±7.5 → 54.5±6.8
Bilirubine (µM)	9.2±4.3 → 1.5±0.6*	15.0±8.7 → 1.5±0.8*	12.9±7.7 → 1.5±0.7†
AST (u/L)	919±519 → 174±116	936±401 → 160±80*	929±420 → 165±89†
ALT (u/L)	558±336 → 43±16	687±375 → 46±30*	640±349 → 45±25†
GGT (u/L)	12.5±9.1 → 3.6±4.7	10.3±6.6 → 0.9±1.7*	11.1±7.2 → 1.7±2.9†
TNF-α (pg/mL)	132±96 → 98.4±60.4	189±118 → 48.5±41.8*	168.5±107 → 66.6±52.7*
NOx (nmol/mL)	74±39 → 127.9±82.4	91±46 → 139.6±68.5	85.5±42.5 → 135.3±69.9*

* p<0.05 respect to basal serum levels,

† p<0.01 respect to basal serum levels.

Laparotomy was performed on all surviving rats 7 days after intraperitoneal bacterial inoculation, and samples were cultured as described above. *E. coli* was isolated in pleural fluid in 1 animal from Group II, and when considering animals included in Group III, 1 animal showed an *E. coli* in peritoneal fluid, 1 rat presented *E. coli* and *Enterococcus* in mesenteric lymph nodes and a third animal had *E. coli* in the liver. The number of surviving rats with positive culture was higher in group II (3/4) than in group III (1/7), although values did not reach statistical significance (p = 0.08).

Histological study of the liver was performed in all surviving rats and showed severe fibrosis in all cases.

## Discussion

To our knowledge, this is the first study to date to assess new therapeutic approaches to reduce mortality during episodes of bacterial peritonitis in cirrhotic rats. These rats develop SBP in the last phases of induction of cirrhosis, but animals are so sick at that time that they usually die during or immediately after the diagnostic paracentesis, becoming an inadequate model for the study of new therapeutic approaches. We developed a new model of induced bacterial peritonitis in rats with cirrhosis, with or without ascitic fluid, and reported mortality rates similar to those found in a clinical setting [15]. This study represents the first application of this animal model to assess new therapeutic options to reduce mortality during episodes of induced bacterial peritonitis.

Our study investigated the effect of TNF-α blockade and/or antibiotics on the inflammatory response and mortality in a cirrhotic rat model with induced bacterial peritonitis. We here report evidences that modulation of inflammatory response, as represented by TNF-α blockade together with the usual third-generation cephalosporin-based therapy in animals with induced bacterial peritonitis, may represent a useful tool to increase survival compared to non-treated rats or treated only with TNF-α blockade. However, this benefit was not statistically significant compared to rats treated only with third-generation cephalosporin. In addition, despite a higher number of positive culture at laparotomy in surviving rats from group II (3/4) than in group III (1/7) (p = 0.08), with the current data we can not speculate about a protective effect of TNF-α blockade with the combined treatment. Probably, additional studies including more animals are required to assess if the association of antibiotic therapy and TNF-α blockade might be a possible approach to reduce mortality in cirrhotic patients with spontaneous bacterial peritonitis.

As pointed out above, higher levels of NO and TNF-α at diagnosis of SBP and during SBP episodes predict complications such as renal insufficiency and survival [7], [11], [19]. Recent findings from our group may offer a clue to explain the maintained inflammatory reaction in SBP. When studying sequential samples of blood from SBP patients under antibiotic therapy, we observed the maintenance of molecular evidence of BT as demonstrated by the presence of bacterial genomic fragments (bacterial DNA) in blood and found that levels kept a direct and significant relation with proinflammatory cytokines and NO [12]. However, in rats, TNF-α blockade appears to blunt hemodynamic disturbances in a model of portal hypertension [13], and reduce episodes of BT in a model of cirrhosis [14]. These data suggest that modulation of the inflammatory response might improve survival, supporting our hypothesis that the use of a selective mAb against TNF-α together with ceftriaxone would decrease mortality in an intraperitoneal infection episode. Since TNF-α is part of the normal immune response against bacterial infections, it is necessary to investigate whether the administration of anti-TNF-α mAb might result in an increased risk of bacterial superinfections. However, in the present study we did not observe superinfections in surviving rats treated with antibiotics and anti-TNF-α mAb.

There were two main analytical findings when comparing samples obtained immediately after i.p. administration of *E. coli* and at laparotomy in surviving rats: first, baseline NOx was the only parameter to show statistically significant differences between surviving and dying rats (Table 1). This information is similar to that reported in patients with SBP [11], and may be related to repeated episodes of BT and stimulation of the immune response prior to i.p. injection with *E. coli*. Indeed, bacteria components such as lipopolysacharide or DNA stimulate the immune response through joining toll-like receptors 4 and 9, respectively [20], [21], and it is likely that higher NOx levels will correlate with more severe haemodynamic disturbances in this model.

Second, TNF-α levels decreased significantly in surviving animals when receiving ceftriaxone alone or in combination with mAb, although values only reached significance in the combination therapy arm. This seems logical when considering the specificity of anti-TNF-α mAb used in this investigation. No differences in the rate of BT were observed when comparing animals included in Groups II or III. These results are similar to others previously reported by our group [14] that showed that anti-TNF-α mAb in non-infected rats with cirrhosis does not increase the likelihood of developing infections. In this investigation, however, rats were infected, and the trend towards an increased persistence of bacteria in mesenteric lymph nodes in animals receiving the combination therapy may point to a decreased ability to fight against infection once it is established. Caution should therefore be recommended when considering the immune modulation with administration of anti-TNF-α mAb in an active infection setting.

TNF-α blockade may be also achieved by several non-monoclonal related molecules. Xanthine derivatives such as pentoxifylline [22] or serotonin 5-hydroxytryptamine (2A) receptor agonists such as 2,5-dimethoxy-4-iodoamphetamine [23] are potent TNF-α inhibitors that might be use to confirm presented data. In addition, the use of these molecules would avoid the formation of anti-drug antibodies.

In conclusion, the administration of ceftriaxone and anti-TNF-α mAb decreases serum TNF-α levels. However, in the present study we did not observe significant differences on survival in cirrhotic rats with induced bacterial peritonitis treated with antibiotics with or without anti-TNF-α mAb. Additional studies including more animals are required to assess if the association of antibiotic therapy and TNF-α blockade might be a possible approach to reduce mortality in cirrhotic patients with bacterial peritonitis, before this therapeutic combination can be recommended. This animal model may represent a useful tool to assess the efficacy of new therapies in these patients.
